# Enhancing maize productivity by mitigating alkaline soil challenges through acidified biochar and wastewater irrigation

**DOI:** 10.1038/s41598-023-48163-9

**Published:** 2023-11-27

**Authors:** Zain ul Shahid, Muqarrab Ali, Khurram Shahzad, Subhan Danish, Sulaiman Ali Alharbi, Mohammad Javed Ansari

**Affiliations:** 1 Department of Agronomy, Muhammad Nawaz Shareef University of Agriculture, Multan, Pakistan; 2 Department of Soil Science, Lasbela University of Agriculture, Water, and Marine Sciences, Uthal, Balochistan Pakistan; 3 Department of Soil Science, Faculty of Agricultural Sciences and Technology, Bahauddin Zakariya University, Multan, Punjab Pakistan; 4 Department of Botany and Microbiology, College of Science, King Saud University, PO Box 2455, 11451 Riyadh, Saudi Arabia; 5 Department of Botany, Hindu College Moradabad (MJP Rohilkhand University Bareilly), Moradabad, 244001 India; 6 Al-Waili Foundation of Science, New York, USA

**Keywords:** Plant sciences, Plant stress responses, Abiotic, Drought

## Abstract

In alkaline soil conditions, the availability of essential nutrients for plant growth becomes limited, posing a significant challenge for achieving optimal maize growth and yield. Exploring the impact of biochar and waste irrigation on soil alkalinity and maize production in arid regions has received limited attention. This study aimed to evaluate the effects of three levels of acidified biochar (0, 5, and 10 Mg ha^−1^) in two growing seasons of maize—spring and autumn. The treatments were applied following a randomized complete block design with three replications. Biochar was applied only in the autumn season, and its residual effects were evaluated in the spring season. The study found that using acidifying biochar at a rate of 10 Mg ha^−1^ significantly increased maize yield by 35.8% compared to no application and by 16.4% compared to a rate of 5 Mg ha^−1^. In the autumn, applying acidified biochar at 10 Mg ha^−1^ reduced soil pH by 3.65% and 6.41% compared to 0 and 5 Mg ha^−1^. In the spring, the same application led to a decrease in soil pH by 5.84% and 7.37% compared to the lower rates. Additionally, using 10 Mg ha^−1^ of acidifying biochar increased soil phosphorus concentration by 87.6% and soil potassium concentration by 38.0% compared to not using biochar, and by 46.2% and 35.0% compared to the 5 Mg ha^−1^ application. These findings suggest that the reduction of soil pH by applying biochar at a rate of 10 Mg ha^−1^ facilitated an increase in nutrient availability in the soil, consequently leading to higher maize yield. Notably, no significant differences were observed in maize productivity and soil properties between the spring and autumn seasons. Therefore, this study paves the way for further exploration into the long-term effects of acidifying biochar on maize productivity and soil properties in similar agroecological contexts.

## Introduction

Soil alkalinity, stemming from an elevated pH level due to the accumulation of soluble salts in arid and semi-arid regions covering approximately 25% of the world's area, poses a significant challenge ^1^. The optimal availability of essential nutrients falls within a narrow pH range of 6 to 7^1^. However, when pH levels exceed this range, it significantly restricts the accessibility of these vital nutrients, leading to a considerable decline in crop yield^2^. Consequently, there is an urgent need for research aimed at developing effective strategies to lower soil pH in these regions. Such research is critical to maximize agricultural productivity and sustainably address the increasing demands for food due to the ever-expanding global population.

Several methods effectively reduce soil pH in alkaline conditions: using organic amendments ^3^, applying elemental sulfur ^4^, using acidifying fertilizers^5^, controlled acid injection^6^, and using pre-formulated soils^6^. However, these strategies have drawbacks: slow results, risk of over-acidification, environmental impact, plant limitations, cost implications, and the need for consistent monitoring, potentially disrupting soil balance^7–9^.

Biochar, when acidified, becomes instrumental in pH reduction in soil ^7^. This specialized form of charcoal derived from organic matter can be intentionally modified to possess acidic properties^8^. When apply to alkaline soil, acidified biochar actively lowers pH levels owing to its inherent acidity^7^. This process contributes to establishing a more harmonized pH environment, particularly advantageous for plants that prefer slightly acidic conditions. The gradual release of acidity by acidified biochar plays a pivotal role in altering and sustaining soil pH levels at the desired range over an extended duration.

Wastewater with a lower pH level is one of the strategies used to reduce the pH of alkaline soils^9,10^. This method operates through various mechanisms: it directly induces soil acidification by introducing acidic elements such as sulfates or nitrates, thus increasing soil acidity through the release of hydrogen ions^9,11^. Additionally, it flushes out acidic compounds, displacing crucial cations, alters soil chemistry through chemical reactions, negatively affects soil microbes by releasing acids, and disrupts nutrient availability^9^. Consequently, this process amplifies the solubility of specific metals while limiting the accessibility of essential nutrients like phosphorus due to the modified pH conditions^10^.

The scientific exploration of acidized field biochar treatments with wastewater in alkaline soil pH lacks in literature. Therefore, our hypothesis revolves around the potential soil pH reduction using acidified biochar with wastewater irrigation. The primary objectives of this study are: i) Characterization of the acidified biochar. ii) Assessment of the influence of acidified biochar on maize productivity over two growing seasons, iii)Evaluation of the effects of acidified biochar on soil properties^12^.

## Materials and methods

### Acidified biochar and wastewater irrigation

The dry poultry dung was pyrolyzed in locally manufactured Klein at temperatures of 350 °C for 2 h ^13^. After pyrolysis, biochar was pulverized to a 2 mm size and kept for cooling. To acidify the biochar, a weak acid solution of citric acid was prepared. Acidification of biochar was done in a ratio of 1:10 (biochar g: acid mL) ^14^. The biochar was mixed with the acid solution in a sealed container, ensuring thorough mixing. It was left to sit for a while before being completely dried. The biochar was characterized as physiochemical (Table 1). Three rates of acidified biochar (0,5 and 10 Mg ha^−1^) were applied. All irrigations were applied using wastewater. The source of wastewater was Wali Muhammad Distributary Multan, Pakistan ^15^. The wastewater was characterized by its chemical properties (Table 1).

**Table 1:** Characteristics of soil, irrigation water, and biochar used in the current study.

Soil characteristics									
Sand (%)	Silt (%)	Clay (%)	Saturation percentage (%)	pH	EC (dS m^−1^)	Organic matter (%)	Total nitrogen (%)	Available phosphorus (mg kg^−1^)	Available potassium (mg kg^−1^)
58	19.5	22.5	38	7.8	2.86	0.58	0.02	13.5	215

Irrigation characteristics									
pH	EC (µS cm^−1^)	Ca^2+^ + Mg^2+^ (meq L^−1^)	Na^+^ (meq L^−1^)	CO_3_^−^ (meq L^−1^)	HCO^3−^ (meq L^−1^)	Cl^−^ (meq L^−1^)	SAR	RSC (meq L^−1^)	
6.9	7128	5.5	65.78	Nil	12.26	4.81	39.47	6.76	

Biochar characterizes									
Nitrogen (%)	Phosphorous (%)	Potassium (%)	Ash (%)	Carbon (%)	Zinc (mg kg^−1^)	Iron (mg kg^−1^)	Manganese (mg kg^−1^)	Boron (mg kg^−1^)	
1.05	0.75	0.35	23.02	61.03	9.04	6.09	4.03	1.02	

*EC* electrical conductivity, *SAR* sodium absorption ration, *RSC* residual sodium carbonate.

### Experimental site

The maize crop was grown in the Experimental Farm of MNS University of Agriculture, Multan, Pakistan 30° 11 ′44″ N and 71° 28 ′31″ E at 129 m above sea level. The soil was sandy loam with 58% sand, 19.5%, and 22.5% clay contents (Table 1). The climate site was arid with low rainfall of less than 250 mm per annum with high temperature.

### Field experiment

In February 2022, maize hybrids YH-1898 was sown on a well-prepared seedbed. The seedbed was thoroughly prepared by subjecting the soil to plowing with a tractor-mounted cultivator for 2–3 rounds, and then, after each pass, planking was done to ensure proper leveling and compaction. The acidified biochar was applied in each plot according to a Randomized Complete Block Design (RCBD) with three replications and maize ridges were created after a single cultivation to mix the material. The crop was sowed using the Choppa method, with a spacing of 20 cm between each plant and 75 cm between each row. The seed rate of 10 kg per acre was applied. The recommended amount of phosphorus (P) and potassium (K), along with one-third of the nitrogen (N), was applied at sowing. The remaining nitrogen was top-dressed in two portions, one at knee height and the other at the blooming stage. During the crop's growth period, weed control in each plot was achieved through a combination of manual hoeing with a hand hoe (khurpa) and the application of a chemical herbicide called gangwei. Irrigations were applied as needed throughout the crop growth stages until the crop achieved physiological maturity. Both canal and tube well water were utilized to irrigate the field. The maize crop was at full maturity.

### Data collection

The stomatal conductance was measured using CIRAS-3-Portable (Company & model) from three tagged plants and the results were averaged. The chlorophyll contents were measured with a chlorophyll meter. The plant height was measured using a measuring scale from five random plants was used. The total dry matter was calculated by biomass sampling from a specified place in each plot. Plants are collected from each treatment and fresh weight of leaves was taken and then dried in the oven and weighed to calculate dry matter. The number of plants was tallied from each treatment, and the cobs per plant were also counted.

The average number of grains per cob was determined by counting the total number of grains on ten randomly selected cobs from each plot, ten cobs were randomly selected from each plot. The total weight of the grains from the selected cobs was measured to determine the average grain weight per cob, and from there, the average grain weight per cob was calculated. Three samples’ of 1000 grains were selected randomly from all the treatments, and their weights were measured. The grain yield from each plot was recorded and converted into Mg ha^−1^. The ten cobs were treated from each replication and their lengths were measured with a measuring scale and their diameters were measured using a vernier scale. Soil samples were sun-dried from the end of the second growing season, autumn. The soil pH, organic matter, soil phosphorus, and potassium were measured using a pH meter, Walkley–Black Method, Olsen’s method and Flame photometer, respectively^12^.

### Statistical analysis

The collected data from soil and maize crops was statistically analyzed using R (R version 4.3.1) software by applying the linear mixed model. The biochar rate was considered a fixed effect while the seasons were considered as random effect. Each season was analyzed independently to check the residual effect of acidified biochar application. The means of treatment were compared using the Tukey multiple comparison test at p < 0.05. The R “emmean” package was used to perform the least square means and adjusted multiple comparison procedures.

### Ethics approval and consent to participate

We all declare that manuscript reporting studies do not involve any human participants, human data, or human tissue. So, it is not applicable.

### Experimental research and field studies on plants (either cultivated or wild), including the collection of plant material, must comply with relevant institutional, national, and international guidelines and legislation

We confirmed that all methods were performed in accordance with the relevant guidelines/regulations/legislation. The seeds were purchased from a local certified seed dealer of the Government of Punjab, Pakistan.

## Results

### Plant height, cob plant^−1^, stomatal conductance, and total dry matter

The 10 Mg ha^−1^ biochar showed higher plant height by 3.48% and 1.92% as compared to 0 and 5 Mg ha^−1^ biochar, respectively (Table 2). Acidified biochar (10 Mg ha^−1^) showed 100 and 50% increase in the cob plant^−1^ compared to 0 and 5 Mg ha^−1^ biochar, respectively . The biochar 5 Mg ha^−1^ showed a 33% increase in the cob plant^−1^ compared to 0 Mg ha^−1^ biochar, respectively. The 10 Mg ha^−1^ biochar showed higher cob plant^−1^ by 67% and 11.33% as compared to 0 and 5 Mg ha^−1^, respectively (Table 2). The 10 Mg ha^−1^ biochar showed 4.60% and 0.46%, increased in the stomatal conductance as compared to 0 and 5 Mg ha^−1^ biochar. The 5 Mg ha^−1^ biochar showed a 4.11% increase in the stomatal conductance compared to 0 Mg ha^−1^, respectively. The 10 Mg ha^−1^ biochar showed 8.14 and 0.61%, increased in the stomatal conductance as compared to 0 and 5 Mg ha^−1^ (Table 2). The 10 Mg ha^−1^ biochar showed higher total dry matter by 10.2% and 9.5% as compared to 0 and 5 Mg ha^−1^ biochar, respectively (Table 2).

**Table 2:** The impact of biochar on plant height, cob per plant, grains per cob, and cob weight grown in spring and autumn seasons with wastewater.

Biochar rate (Mg ha^−1^)	Plant height (cm)	Cob plant^−1^	Stomatal conductance	Total dry matter
Spring 2022				
0	180.67 ± 1.53a	1.00 ± 0.01a	14.00 ± 3.61a	147.61 ± 26.69
5	187.33 ± 4.04a	1.67 ± 0.58a	22.33 ± 5.51a	141.47 ± 20.4
10	187.33 ± 3.06a	1.67 ± 0.58a	29.33 ± 10.07a	168.37 ± 40.77
Autumn 2022				
0	182.67 ± 2.89ab	1.00 ± 0.01	14.36 ± 7.23a	148.43 ± 17.5a
5	187.33 ± 2.08ab	1.33 ± 0.58	21.33 ± 7.23a	167.83 ± 46.01a
10	190.00 ± 1.00b	2.00 ± 0.01	31.00 ± 8.89a	133.38 ± 6.47a

Values are the mean and standard deviation (n = 3). Values with the same letters within the season are statistically not significant at p < 0.05.

### Cob diameter, cob length, SPAD value

In autumn acidified biochar (10 Mg ha^−1^) showed 7.79 and 2.64%, increased in the cob diameter as compared to 0 and 5 Mg ha^−1^ biochar application (Table 3). In autumn, acidified biochar (5 Mg ha^−1^) showed a 4.9% increase in the cob diameter under canal water compared to control, respectively. In spring acidified biochar at rate of 5 Mg ha^−1^ showed 3.2 and 7.08%, increased in the cob diameter as compared to 0 and 10 Mg ha^−1^ biochar application. In spring acidified biochar at rate of 10 Mg ha^−1^ showed a 3.7% increase in the cob diameter water compared to control, respectively. The biochar at rate of 5 Mg ha^−1^ showed higher cob diameter by 3.47% and 5.39% as compared to 10 Mg ha^−1^ and control, respectively. The biochar at rate of 10 Mg ha^−1^ showed higher cob diameter by 1.85% as compared to 0 Mg ha^−1^ biochar (Table 3). In autumn acidified biochar (10 Mg ha^−1^) showed 4.60 and 0.46%, increased in the cob length as compared to control and 5 Mg ha^−1^ biochar . In autumn, 5 Mg ha^−1^ acidified biochar showed a 4.11% increase in the cob length compared to the 0 Mg ha^−1^, respectively. In spring 10 Mg ha^−1^ acidified biochar showed 8.14% and 0.61%, increases in the cob length as compared to control and 5 Mg ha^−1^ biochar. In spring 5 Mg ha^−1^ biochar showed a 7.48% increase in the cob length compared to the control. The 10 Mg ha^−1^ biochar showed higher cob length by 5.86% and 0.39% as compared to the 0 and 5 Mg ha^−1^ biochar, respectively. The 5 Mg ha^−1^ biochar showed a higher cob length by 5.53% as compared to the control (Table 3). In the spring season, 10 Mg ha^−1^ biochar increased SPAD value compared to control and biochar (5 Mg ha^−1^). In the autumn season 10 Mg ha^−1^ biochar showed 2.75 and 1.02%, increases in the SPAD value as compared to 0 and 5 Mg ha^−1^ biochar. The 5 Mg ha^−1^ biochar showed a 1.70% increase in the SPAD value compared to 0 Mg ha^−1^, respectively. The 10 Mg ha^−1^ biochar showed higher SPAD values by 0.12% and 0.087% as compared to the 0 and 5 Mg ha^−1^, respectively (Table 3).

**Table 3:** The impact of biochar on cob diameter and cob length grown in spring and autumn seasons with wastewater.

Biochar rate (Mg ha^−1^)	Cob diameter (mm)	Cob length (cm)	SPAD value
Spring 2022			
0	41.34 ± 1.38a	18.04 ± 0.14a	56.57 ± 3.88a
5	44.27 ± 0.76a	19.39 ± 0.28a	59.77 ± 3.84a
10	42.87 ± 3.67a	19.51 ± 0.54a	60.63 ± 2.72a
Autumn 202			
0	41.71 ± 0.98a	18.45 ± 0.51a	60.27 ± 1.31a
5	43.78 ± 1.07a	19.22 ± 0.37a	61.3 ± 4.73a
10	44.96 ± 1.06a	19.3 ± 0.50 a	61.93 ± 4.74a

Values are the mean and standard deviation (n = 3). Values with the same letters within the season are statistically not significant at p < 0.05.

### Grain yield and grain per cob

**Figure 1:**
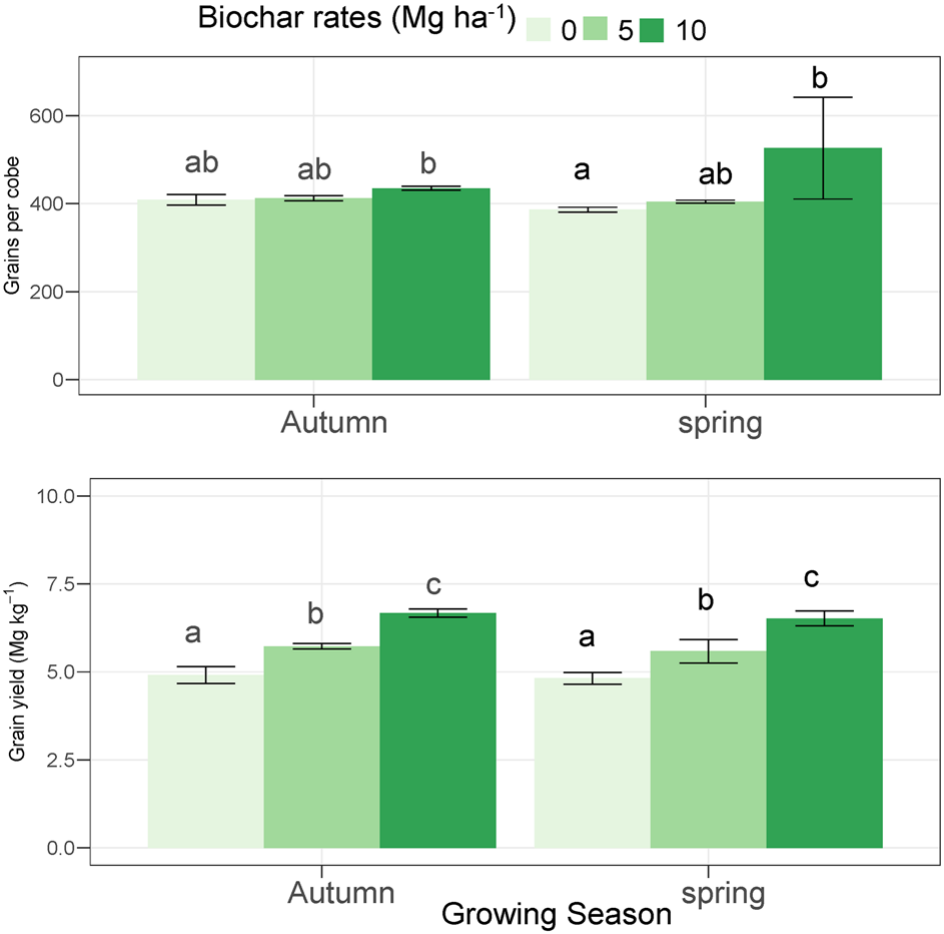
The impact of different levels of acidified biochar on the grain yield and grains per cob in two growing seasons of maize crop during 2022. The error bars show the standard deviation (n = 3). The bars with the same letters are statistically non-significant at p < 0.05.

The 10 Mg ha^−1^ biochar showed higher grain yield by 35.84% and 16.40% as compared to 0 and 5 Mg ha^−1^ biochar, respectively. The biochar (10 Mg ha^−1^) showed higher grain yield by 35.80% and 16.60% as compared to the control and 5 Mg ha^−1^, respectively (Fig. 1). The 10 Mg ha^−1^ biochar showed increase of 6.35 and 5.49%, grain cob^−1^ as compared to 0 and 5 Mg ha^−1^ biochar. The 5 Mg ha^−1^ Biochar showed a 0.81% increase in grain cob^−1^ under compared to 0 Mg ha^−1^ (Fig. 1). The 10 Mg ha^−1^ biochar showed a higher cob^−1^ by 7.36% and 4.35% as compared to the 0 and 5 Mg ha^−1^ biochar, respectively. The 5 Mg ha^−1^ biochar showed a higher number of grain cob^−1^ by 2.88% as compared to 0 Mg ha^−1^.

### 1000-grain weight and cob weight

**Figure 2:**
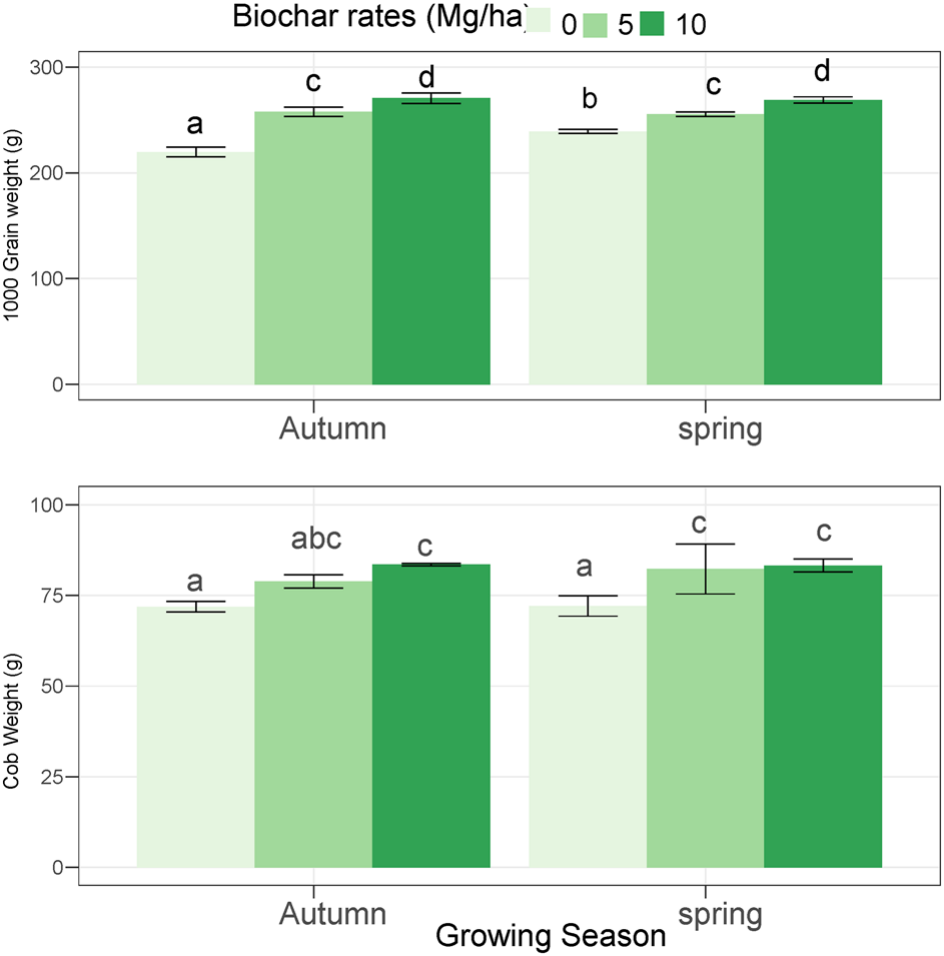
The impact of different levels of acidified biochar on the cob weight and 1000 grain weight in two growing seasons of maize crop during 2022. The error bars show the standard deviation (n = 3). The bars with the same letters are statistically non-significant at p < 0.05.

The 10 Mg ha^−1^ biochar showed a higher 1000-grain weight by 23.84% and 4.99% as compared to 0 and 5 Mg ha^−1^ biochar, respectively (Fig. 2). The 10 Mg ha^−1^ biochar showed higher 1000-grain weight by 17.61% and 5.14% as compared to the 0 and 5 Mg ha^−1^, respectively. The control treatment showed a minimum grain weight per cob while the biochar applied at 10 Mg ha^−1^ showed higher grain weight per cob by 16.12% and 5.88% as compared to 0 and 5 Mg ha^−1^ biochar applied, respectively. The 10 Mg ha^−1^ biochar showed higher grain weight cob^−1^ by 15.34% and 4.60% as compared to 0 and 5 Mg ha^−1^, respectively (Fig. 2).

### pH, organic matter, phosphorous and potassium contents

The application of acidified biochar (5 and 10 Mg ha^−1^) showed a decrease in soil pH as compared to control. The soil pH was decreased with increase in rate of acidified biochar. The higher response in soil pH decrease was seen with higher rate biochar. In the autumn season, acidified biochar application at the rate of 10 Mg ha^−1^ showed a decrease in pH by 3.65 and 6.41 as compared to 0 and 5 Mg ha^−1^ biochar, respectively while in the spring season, acidified biochar application at the rate of 10 Mg decreased soil pH by 5.84% and 7.37% as compared to 0 and 5 mg ha^−1^ biochar, respectively (Table 4). The soil organic matter was increased with the application of acidified biochar. The increase in organic matter in both seasons was inconsistent. The higher organic matter increase was seen in spring 2022 with a rate of 10 Mg ha^−1^. The application of acidified biochar at rates of 10 and 5 Mg ha^−1^ showed increases of 26.39 and 56.94% compared to the control in the spring growing season. In the autumn, the application of acidified biochar at rates of 10 and 5 Mg ha^−1^ showed increases of 16.25 and 32.50% compared to the control, respectively (Table 4). The biochar application significantly improved the soil phosphorus contents in both the spring and autumn seasons. The soil phosphorus contents increase with an increase in the rate of acidified biochar. The acidified biochar at a rate of 10 Mg ha^−1^ showed higher phosphorus contents in both growing seasons, while the lowest phosphorus contents were seen in the plots where no biochar was applied. The acidified biochar at rates of 5 and 10 Mg ha^−1^ showed an increase in phosphorus contents by 28.3% and 87.5% over no biochar (Table 4). The higher potassium contents were seen with the application of acidified biochar at a rate of 10 Mg ha^−1^ in both growing seasons while the lowest potassium contents were seen in control plots where no biochar was applied. The acidified biochar at rates of 5 and 10 Mg ha^−1^ showed increased phosphorus contents by 6.43% and 30.04% over 0 Mg ha^−1^ biochar (Table 4).

**Table 4:** The impact of acidified biochar on soil pH, organic matter, phosphorus contents, and potassium after two season of maize crop grown in the spring and autumn season utilizing wastewater irrigation.

Biochar rate	pH	Organic matter	Phosphorous content	Potassium content
Spring 2022				
0	8.14 ± 0.33a	0.72 A ± 0.07a	5.4 ± 1.04a	164.00 ± 4.36a
5	7.86 ± 0.27a	0.91 A ± 0.02ab	6.93 ± 2.57ab	167.67 ± 7.51a
10	7.65 ± 0.36a	1.13 A ± 0.07b	10.13 ± 3.33c	226.33 ± 10.21b
Autumn 2022				
0	8.160 ± 0.33a	0.8 A ± 0.07a	7.27 ± 3.83a	155.33 ± 16.17a
5	7.716 ± 0.36a	0.93 A ± 0.12ab	7.20 ± 2.5ab	165.33 ± 14.57a
10	7.601 ± 0.33a	1.06 A ± 0.11b	12.27 ± 3.72bc	202.00 ± 19.08b

Values are the mean and standard deviation (n = 3). Values with the same letters within the season are statistically not significant at p < 0.05.

## Discussion

The present field study aimed to assess the impact of varying acidified biochar rates (0, 5, and 10 Mg ha^−1^) on maize productivity and soil properties across two growing seasons (Autumn and Spring). This investigation took place in the research field of MNS-University of Agriculture, Multan, Pakistan, utilizing wastewater for irrigation.

The combined application of acidified biochar and wastewater irrigation moderately reduced the soil pH during both growing seasons. The decreased in soil pH by wastewater might be due to nitrification of ammonium and oxidation of organic compounds. Rusan et al. ^9^ found decreased in soil pH after two years application of wastewater. However, after two years the soil pH was increased in their study that might be due to high contents of Na, Ca, and Mg in wastewater^9^. Mohammad and Mazahresh ^10^ found that with increasing the wastewater amount, the pH of soil decreased. The wastewater lower down pH by 2.7% at 0–30 cm soil depth with higher wastewater amount. The higher contents of ammonium in the wastewater resulted its accumulation in the soils. The hydrogen ions released as the results of nitrification of ammonium which caused the soil pH to lower down^10^. The high soil buffering capacity might be retain the original pH after sometimes. Therefore, pH might be expected to rise again after some times. Hayes et al. ^11^ found decreased in pH from 7.9 to 7.6 with application of effluent water in turf grass field. The pH decreased was attributed to nitrification of added nitrogen sources.

The acidified biochar also played their role in lower down the soil pH as reported in many literatures. Abd El-Mageed et al. ^16^ found that 10 and 5 t ha^−1^ biochar decreased pH by 5.1% and 1.9%, respectively as compared to 0 t ha^−1^, respectively. Ahmed et al. ^17^ found that acidified green waste biochar and corn cob biochar showed decreased in soil pH as compared to no biochar, green waste biochar and corn cob biochar. In another study, Ippolito et al. ^18^ found initially increased in soil pH with acidic biochar after one month of application but the pH went down in 4 to 6 months after application.

The lower down of pH by wastewater irrigation and acidified biochar resulted in increase of soil organic matter, soil phosphorus and potassium concentration (Table 4). Rusan et al. ^9^ found increased in the soil nitrogen, phosphorus, and potassium concentration with the waster irrigation over a period of ten years application in forage crop cultivation. These might be attributed due to accumulation of wastewater nutrients in the soil. In another study, Mohammad et al.^10^.

found increase of 89.1% in soil available phosphorus with wastewater application as compared to potabile water in forage crops. The soil phosphorus and potassium concentration were increased with application of acidified biochar in many studies. Ippolito et al. ^18^ found increased the soil phosphorus concentration with application of acidic biochar. The soil concertation of phosphorus increased with increase of acidic biochar rate and time duration. The 10% biochar rate showed a higher concentration of soil phosphorus over a 10-month duration. In another study, Abd-El-Maged et al. ^16^ found 34.4% and 24.8% increase in phosphorus concentration with the application of 5% and 10% acidified biochar as compared to 0% acidified biochar. They also recorded a 20.5% and 35.8% increase in potassium concentration over 0% acidified biochar. The low pH, total porosity, water holding capacity, bulk density, and organic matter contents of soil might have contributed to the increase in the supply of macronutrients in soil.

**Figure 3:**
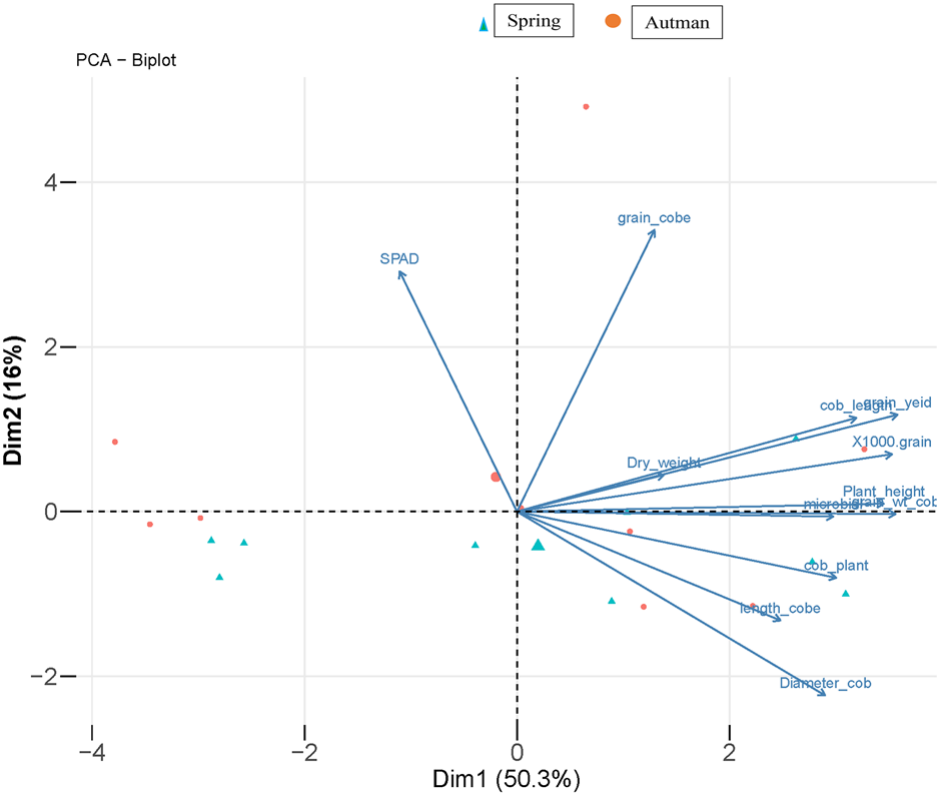
The PCA biplot analysis showing the relationships among the different measured parameters.

**Figure 4:**
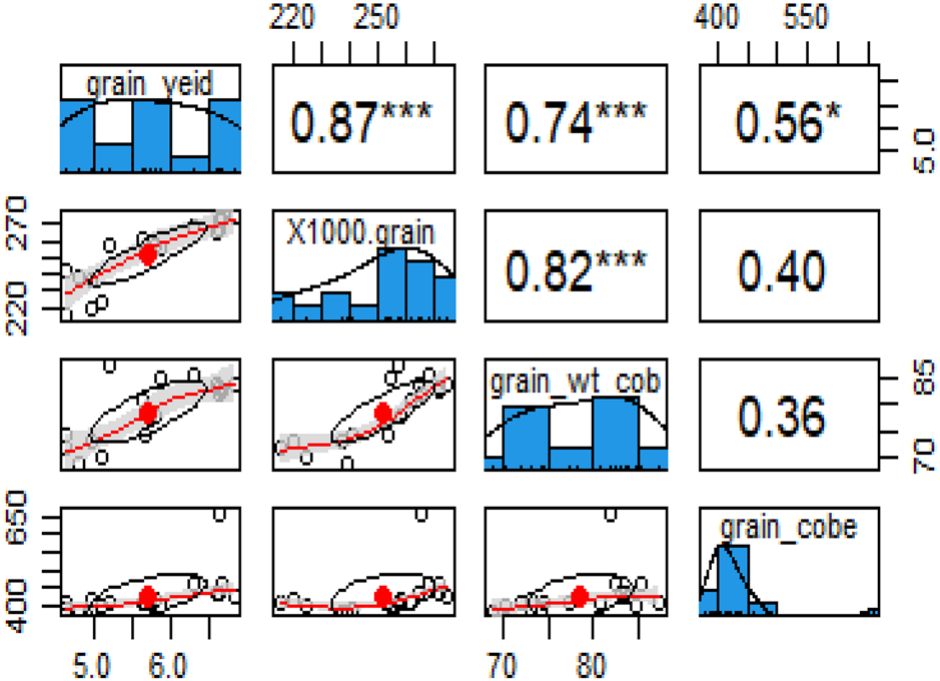
Correlation matrix among grain yield, 100-grain weight, grain weight per cob, and grain per cob.

The maize growth, yield, and physiological attributes showed a positive response to acidified biochar under wastewater irrigation in both growing seasons. This might be due to a decrease in soil pH and an increase in the availability of soil phosphorus and potassium contents (Table 4). Ahmed et al.^17^ found an increase in plant height, cob length, and cob diameter with the application of acidified green waste biochar and acidified corn cob biochar as compared to simple green waste biochar and corn biochar at 75%, and 100% phosphorus rates. In another study, Mousavi and Mousavi ^19^ found an increase in stem height, 100-grain weight, and grain yield when treated with 100% treated wastewater as compared to well water. Positive correlation among the plant parameters were observed, as depicted in Figs. 3 and 4. This indicates that an increase in growth attributes led to a corresponding increase in the yield parameters.

## Conclusion

The combined application of acidified biochar and wastewater led to a moderate reduction in soil pH across growing seasons, attributed to processes like ammonium nitrification and organic compound oxidation. Both wastewater irrigation and acidified biochar contributed to increased soil organic matter, phosphorus, and potassium concentrations, positively impacting soil nutrient availability. The maize growth, yield, and physiological attributes demonstrated a positive response to acidified biochar under wastewater irrigation, likely influenced by enhanced soil phosphorus and potassium availability. These findings highlight the potential of acidified biochar and wastewater irrigation to positively influence soil properties and maize productivity, suggesting promising agricultural practices for sustainable crop growth and soil improvement.

## Data Availability

All data generated or analysed during this study are included in this published article.

## References

[1] Riaz MU (2020). Fate of micronutrients in alkaline soils. Fate Micronutr. Alkaline Soils.

[2] Emran, M., Rashad, M., Gispert, M. & Pardini, G. Increasing soil nutrients availability and sustainability by Glomalin in alkaline soils. *Int. J. Agricult. Biosyst. Eng.* **2**. http://www.aascit.org/journal/ijabe (2017).

[3] Dibner JJ, Buttin P (2002). Use of organic acids as a model to study the impact of gut microflora on nutrition and metabolism. J. Appl. Poultry Res..

[4] Ali Turan M, Taban S, Vahap Katkat A, Kucukyumuk Z (2005). The evaluation of the elemental sulfur and gypsum effect on soil pH, EC, SO_4_-S and available Mn content. J. Food Agric Environ..

[5] Vašák F, Černý J, Buráňová Š, Kulhánek M, Balík J (2015). Soil pH changes in long-term field experiments with different fertilizing systems. Soil Water Res..

[6] Faisal, A. A. H. & Hussein, A. A. An acidic injection well technique for enhancement of the removal of copper from contaminated soil by electrokinetic remediation process. *Sep. Sci. Technol.* **50**, 2578–2586 (2015).

[7] Doydora SA (2011). Release of nitrogen and phosphorus from poultry litter amended with acidified biochar. Int. J. Environ. Res. Public Health.

[8] Lehmann J, Joseph S (2015). Biochar for environmental management: An introduction. Biochar Environ. Manag..

[9] Mohammad Rusan, M. J., Hinnawi, S. & Rousan, L. Long term effect of wastewater irrigation of forage crops on soil and plant quality parameters. *Desalination* **215**, 143–152 (2007).

[10] Mohammad MJ, Mazahreh N (2003). Changes in soil fertility parameters in response to irrigation of forage crops with secondary treated wastewater. Commun. Soil Sci. Plant Anal..

[11] Hayes, A. R., Mancino, C. F. & Pepper, I. L. Irrigation of turfgrass with secondary sewage effluent: I. Soil and leachate water quality. *Agron. J.* **82**, 939–943 (1990).

[12] Chintala R, Mollinedo J, Schumacher TE, Malo DD, Julson JL (2013). Effect of biochar on chemical properties of acidic soil. Arch. Agron. Soil Sci..

[13] Shahzad K, Abid M, Sintim HY (2018). Wheat productivity and economic implications of biochar and inorganic nitrogen application. Agron. J..

[14] ur Rehman, M. Z. *et al.* Effect of acidified biochar on bioaccumulation of cadmium (Cd) and rice growth in contaminated soil. *Environ Technol Innov* **19**, 31 (2020).

[15] Abd El-Mageed TA (2021). Acidified biochar as a soil amendment to drought stressed (*Vicia faba* L.) plants: Influences on growth and productivity, nutrient status, and water use efficiency. Agronomy.

[16] Abd El-Mageed TA (2021). Acidified biochar as a soil amendment to drought stressed (*Vicia faba* L.) plants: Influences on growth and productivity, nutrient status, and water use efficiency. Agronomy.

[17] Ahmed, N. *et al.* Effect of acidified biochar on soil phosphorus availability and fertilizer use efficiency of maize (*Zea mays* L.). *J. King Saud Univ. Sci.* **33**, 81 (2021).

[18] Ippolito JA, Ducey TF, Cantrell KB, Novak JM, Lentz RD (2016). Designer, acidic biochar influences calcareous soil characteristics. Chemosphere.

[19] Roholla Mousavi S, Shahsavari M (2014). Effects of treated municipal wastewater on growth and yield of maize (*Zea mays*). Biol. Forum.

