# Author Correction: Enhancing maize productivity by mitigating alkaline soil challenges through acidified biochar and wastewater irrigation

**DOI:** 10.1038/s41598-023-49412-7

**Published:** 2023-12-18

**Authors:** Zain ul Shahid, Muqarrab Ali, Khurram Shahzad, Subhan Danish, Sulaiman Ali Alharbi, Mohammad Javed Ansari

**Affiliations:** 1 Department of Agronomy, Muhammad Nawaz Shareef University of Agriculture, Multan, Pakistan; 2 Department of Soil Science, Lasbela University of Agriculture, Water, and Marine Sciences, Uthal, Balochistan Pakistan; 3 Department of Soil Science, Faculty of Agricultural Sciences and Technology, Bahauddin Zakariya University, Multan, Punjab Pakistan; 4 Department of Botany and Microbiology, College of Science, King Saud University, PO Box 2455, 11451 Riyadh, Saudi Arabia; 5 Department of Botany, Hindu College Moradabad (MJP Rohilkhand University Bareilly), Moradabad, 244001 India; 6 Al-Waili Foundation of Science, New York, USA

Correction to: *Scientific Reports* 10.1038/s41598-023-48163-9, Published online 27 November 2023

The original version of this Article contained an error in the Acknowledgements section, where the project number was incorrect.

“This project was supported by Researchers Supporting Project number (RSP2023R385), King Saud University, Riyadh, Saudi Arabia.”

now reads:

“This project was supported by Researchers Supporting Project number (RSP2023R5), King Saud University, Riyadh, Saudi Arabia.”

The original Article has been corrected.

